# Computationally efficient mutual authentication protocol for remote infant incubator monitoring system

**DOI:** 10.1049/htl.2018.5006

**Published:** 2019-07-09

**Authors:** Subramani Jegadeesan, Muneeswaran Dhamodaran, Maria Azees, Swaminathan Sri Shanmugapriya

**Affiliations:** 1 Department of Electronics and Communication Engineering, M. Kumarasamy College of Engineering, Karur, Tamilnadu, India; 2 Department of Electronics and Communication Engineering, GMR Institute of Technology, Rajam, Andra Pradesh, India; 3 Department of Electronics and Communication Engineering, V.S.B. Engineering College, Karur, Tamilnadu, India

**Keywords:** Internet of Things, wireless sensor networks, public key cryptography, message authentication, cryptography, health care, protocols, patient monitoring, cloud computing, cryptographic protocols, telecommunication security, resource-limited sensors, continuous operations, accurate operations, received information, authenticity, doctors, computationally efficient mutual authentication protocol, incubator monitoring systems, computational cost, existing protocol, remote infant incubator monitoring system, cloud computing, wireless medical sensor networks, remote healthcare monitoring, healthcare monitoring system

## Abstract

Internet of Things (IoT), cloud computing and wireless medical sensor networks have significantly improved remote healthcare monitoring. In a healthcare monitoring system, many resource-limited sensors are deployed to sense, process and communicate the information. However, continuous and accurate operations of these devices are very important, especially in the infant incubator monitoring system. Because important decisions are made on the received information. Therefore, it is necessary to ensure the authenticity between the incubator monitoring system and doctors. In this work, a public key encryption based computationally efficient mutual authentication protocol is proposed for secure data transmission between incubator monitoring systems and doctors or administrators. The proposed protocol improves performance and reduces the computational cost without compromising the security. The security analysis part shows the strength of the proposed protocol against various attacks, performance analysis part shows that the proposed protocol performs better than other existing protocol based on Rivest–Shamir–Adleman and elliptic-curve cryptography schemes.

## Nomenclature

### Symbol

$\delta $
security parameters
$P_{{\rm sys}}$
system parameters
$\lpar PRI\comma \; PUB\rpar $
private and public keys
$\lpar msg\rpar $
original message
$\lpar CT\rpar $
ciphertext
$msg_{\max }$
maximum length of the message
*A* and *B*: users in the system
$e_{PUB_A}$, $e_{PUB_B}$
encryption process using users public key
$d_{PRI_A}\comma \; d_{PRI_B}$
decryption process using users private key
$\overline {msg_A} \comma \; \overline {msg_B} $
received messages after decryption

## 1 Introduction

In the European countries, women, those who are working up to 40 h and above in a week and those who are travelling long distances to work, they are giving birth to premature babies [1]. Also, babies are having more feeding and breathing problems. A baby born before 37 weeks is called a premature baby. In the US, about one in ten babies is born before 37 weeks in each year [2, 3]. Early born babies may easily get affected by a lot of health issues [4]. Therefore, they need to stay longer days in the hospital than normal babies. However, some premature babies need to take care of using the incubator system in the hospital to avoid health issues. In recent days, due to the development of advanced technologies, more several premature babies are surviving than the early days.

In the hospitals, the doctors and nurses are required to monitor the premature babies in the incubator all the time manually [1, 2], i.e. the presence of a doctor or a nurse is always required and they need to continuously monitor the health status of an infant in the incubator [5–7]. However, doctors and nurses need to do other works in the hospital at the same time. So, there is a need for efficient infant incubator monitoring system to overcome the above problems by using a wireless medical sensor network [8–10]. It is capable of collecting and transmitting medical information to the doctors and relatives, who are in a remote location. However, data transmission through wireless medical sensor networks has affected by many security problems [11–13], such as data security (data integrity, data usability, data auditing) and privacy (patient information). Therefore, it is necessary to provide secure data transmission and user authentication in a resource-limited environment.

The main objective of the proposed infant incubator monitoring system is

- To propose a new encryption scheme and use it to construct a computationally efficient mutual authentication protocol.
- To provide secure data transmission between the incubator system and doctors or administrators in the hospital to avoid replay attack, impersonation attack and man in the middle attack.
- Implementation of a remote infant incubator monitoring system that primarily addresses continuous physical monitoring can significantly reduce doctors and nurses’ participation and enable doctors and families to access baby health information anytime and anywhere securely.

The rest of this Letter is organised as follows: Section 2 describes the related works. The preliminaries are described in Section 3. The proposed remote incubator monitoring system presented in Section 4. Section 5 analyses the computational cost. Section 6 describes the security analysis. Section 7 describes the public-key and ciphertext size optimisation. Section 8 presents the performance analysis and results of our proposed scheme with existing schemes. Section 9 gives the conclusion of our work.

## 2 Related works

Many of the infant incubator systems only show the temperature and other important parameter in the display, which is fixed in the incubator system [4]. The collected information from the sensor cannot transfer to the remote places and the incubator system cannot control from the remote places by using the existing system.

In the United States, New Orleans hospital introduced a new mobile application to monitor the premature babies in the incubator system by the parent and their relatives through the web cameras fixed in the incubator [14]. Through this system, parents can see their babies and also they can monitor important parameters that affect the early born babies. The early born babies need to spend one week to one month in an incubator system for normal growth and to avoid other health problems. Some of the existing incubator systems that measure the temperature, humidity and pulse rate and it is transferred to the doctors and parent through the mobile application [15]. Also, it will give the emergency alarm to the doctors and parent in case of an abnormal condition.

At the hospital, doctors can check the health status of the infant by using wireless medical sensor networks. Body temperatures, blood pressure, heartbeat, pulse rate and sugar level are the important parameters used to check the health status of the infant [16]. In recent days, researchers working towards to develop the wireless sensor network based health monitoring system. There are lots of challenges when transferring medical data through wireless medical sensor networks. The challenges are reliable data transmission, time of data delivery, secure data transmission, power management and so on, one of the important challenges in the wireless medical sensor network is data security, and it will affect the infant privacy [17]. If health details of the infant are leaked or eavesdropped by intruders, then it will create problems for the infant and doctors. Thus the use of infant health details by intruders can cause life-threatening risks to the infant and make the infant private matters publicly available [18].

## 3 Preliminaries

### 3.1 Internet of Things (IoT) in medical applications

**Fig. 1:**
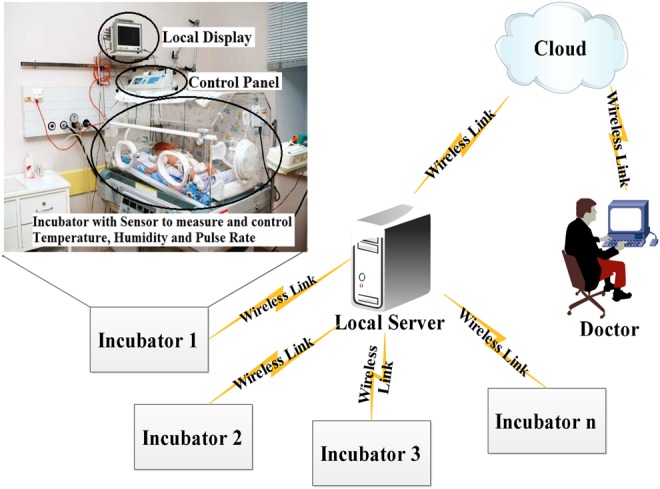
System model

IoT-based medical applications depend on different types of medical sensor devices and modern technologies, as shown in Fig. 1. For example, the medical information of the baby in the incubator is measured and it is communicated to remote users. IoT-based wireless medical sensor networks consist of sensor devices, cloud server and users.

*Medical sensor devices:* Resource-limited devices which collect the information from the infant incubator system and it is communicated to the cloud server. But, to create a secure channel between sensor devices and cloud server, the medical sensor devices need to authenticate. Otherwise, the transferred information from the sensor is not trusted.
*Cloud server:* Receives data from the medical sensor devices and it gives the services to the authorised user after the data processing. To avoid the monitoring of medical information by the unauthorised users, the server needs to authenticate.
*Users:* Persons who get the services form the cloud server for monitoring the infant incubator system and also to control the incubator system.

## 4 Proposed system

The proposed remote infant incubator monitoring system mainly consists of various sensors to measure values of important parameters of premature babies. The incubator system used to monitor and control the temperature, humidity level and pulse rate of babies in the incubator. The system is designed to send all the sensed information from the incubator to doctors, parents and their relatives. Similarly, doctors can send control information to incubators in a secure manner using IoT.

In an IoT environment, the energy limited wireless medical sensors are interacting with central servers. However, the energy limited sensors need to transmit a large amount of data and it should produce high efficiency with a reasonable computational cost. Therefore the proposed computationally efficient mutual authentication protocol is used to provide better data security as well as performance.

In this proposed scheme, a new encryption scheme and mutual authentication protocol was developed for the energy limited wireless medical sensor networks and low power networks that are implemented in remote infant incubator monitoring system. The proposed encryption scheme permits medical sensors to do a large number of computations efficiently with less time. The new encryption scheme based mutual authentication protocol performs well in terms of usability, ciphertext size and efficiency when compared to other conventional authentication schemes.

The proposed encryption scheme converts the original message into a ciphertext size of 274 bits, but the conventional encryption scheme gives the ciphertext with the size of 1172 bits. The ciphertext size is 898 bits lesser than the conventional encryption scheme. Also, the developed encryption scheme does not require the support of the trusted third party during the system initialisation. The four main modules of the proposed system can be described below:

(a) system setup algorithm,
(b) key generation algorithm,
(c) encryption algorithm and
(d) decryption algorithm.

(a) *System setup algorithm:* It takes the important security parameter $\delta $ as an input and it gives the system parameter $P_{{\rm sys}}$ as an output.
(b) *Key generation algorithm:* It takes the output of the system setup algorithm as an input and it generates the private and public keys as an output $\lpar PRI\comma \; PUB\rpar $.
(c) *Encryption algorithm:* It converts the original message $\lpar msg\rpar $ into a ciphertext $\lpar CT\rpar $ by using the receiver's public key $\lpar PUB\rpar $.
(d) *Decryption algorithm:* It converts ciphertext $\lpar CT\rpar $ into an original message $\lpar msg\rpar $ by using the receiver's private key $\lpar PRI\rpar $.

Similarly, the proposed mutual authentication protocol consists of four important algorithms described as follows:

(a) system setup algorithm,
(b) key generation algorithm,
(c) initialisation algorithm and
(d) authentication algorithm.

### 4.1 Possible attacks on the mutual authentication protocol

The main focus of this protocol is to check the validity of participating users. The opponent may have different goals against mutual authentication protocol. The possible attacks on a mutual authentication protocol are

*Replay attack:* A potential eavesdropper records the communication between two users and it replays the recorded message to the particular user.
*Impersonation attack:* An opponent successfully assumes the uniqueness of one of the genuine users in the communication.
*Man in the middle attack:* An opponent secretly transmits the message or alters the communication between the users. But the users believing that they are connected directly with each other.

### 4.2 Computationally efficient encryption algorithm

The proposed computationally efficient encryption algorithm consists of four modules, (a) system setup algorithm, (b) key generation algorithm, (c) encryption algorithm and (d) decryption algorithm.

- *System setup algorithm:* The algorithm takes the security parameters ‘$\delta $’ as an input; it selects the database as $m\comma \; n\comma \; o\comma \; x\comma \; y\comma \; {\bi z}$ and $msg_{\max }$
  where *m*, *n* are the co-primes, $msg_{\max }$ is the maximum length of the message, *y* is the number of dimensions and ***z*** is the public key vector.
  The system setup algorithm generates system parameters $P_{sys}$ as output $m\comma \; n\comma \; o\comma \; x\comma \; y\comma \; {\bi z}$ and $msg_{\max }$.
- *Key generation algorithm:* This algorithm used to generate private and public keys $\lpar PRI\comma \; PUB\rpar $ by using system parameter $P_{{\rm sys}}$ as an input.
  o *Step 1:* It selects the *n*-dimensional vector $\lpar k_1\comma \; k_2\comma \; \ldots \comma \; k_n\rpar $ randomly, i.e. $k_i \in \lpar 1\comma \; 2\comma \; 3\comma \; \ldots \comma \; m - 1\rpar $, and $i = \lpar 1\comma \; 2\comma \; 3\comma \; \ldots \comma \; n\rpar $. The value of $k_i$ should satisfies $gcd\lpar k_n\comma \; m\rpar = 1$.
  o *Step 2:* A public key *PUB* consists of ***z*** vectors, i.e.
    (1) $$PUB_l = \lpar PUB_{l\comma 1}\comma \; PUB_{l\comma 2}\comma \; \ldots \comma \; PUB_{l\comma n}\rpar \eqno\lpar 1\rpar $$
    where $l = \lpar 1\comma \; 2\comma \; 3\comma \; \ldots \comma \; z\rpar $.

The value of $PUB_{l\comma n}$ can be computed as follows:

If the value of $l = 1$, then it calculates the value $PUB_{l\comma n}$ as

(2) $$PUB_{l\comma n} = k_n^{ - 1} \lpar 1 - k_1PUB_{1\comma 1} + k_2PUB_{1\comma 2}\comma \; \ldots \comma \; k_{n - 1}PUB_{1\comma n - 1}\rpar \eqno\lpar 2\rpar $$

(3) $$\eqalign{PUB_{l\comma n} & =k_n^{ - 1} \lpar PUB_{l - 1\comma n} + s_lt - \lpar k_1PUB_{l\comma 1} + k_2PUB_{l\comma 2} \cr & \quad + \cdots + k_{n - 1}PUB_{l\comma n - 1}\rpar \rpar } \eqno\lpar 3\rpar $$

Consider the private and public keys as

(4) $$\lpar PRI\comma \; PUB\rpar = \lpar \lpar k_1\comma \; k_2\comma \; ...\comma \; k_n\rpar \comma \; \lpar PUB_1\comma \; PUB_2\comma \; ...\comma \; PUB_z\rpar \rpar \eqno\lpar 4\rpar $$

- *Encryption algorithm:* The encryption algorithm uses the receiver public key *PUB* to encrypt the original message $\lpar msg\rpar $. The length of the message should be $msg = msg_{\max }$.
  The algorithm randomly chooses the integer *l* and the value of the integer is $2 \le l \le z$.
  The ciphertext
  (5) $$CT = \lpar PUB_{l\comma 1}\comma \; PUB_{l\comma 2}\comma \; \ldots \comma \; PUB_{l\comma n}\rpar \eqno\lpar 5\rpar $$
  and
  (6) $${\rm the}\, {\rm value}\, \gamma = PUB_{l - 1\comma n}.\eqno\lpar 6\rpar $$
  Again, it chooses the random integer *l* and computes the ciphertext as
  (7) $$CT = CT + PUB_{l\comma \gamma } = \gamma + PUB_{l - 1\comma n}\eqno\lpar 7\rpar $$
  The algorithm repeats the procedure for the $x - 1$ times and computes the ciphertext as
  (8) $$CT = CT + \lpar msg - \gamma \rpar PUB_1\bmod \lpar m\rpar \eqno\lpar 8\rpar $$
- *Decryption algorithm:* It takes the ciphertext $CT = \lpar ct_1\comma \; ct_2\comma \; \ldots \comma \; ct_n\rpar $, which is generated by using the receiver's public key
  (9) $$PUB = \lpar PUB_1\comma \; PUB_2\comma \; \ldots \comma \; PUB_z\rpar \eqno\lpar 9\rpar $$
  Once, the cipher text received by the receiver then it is decrypted by using a receiver private key
  (10) $$PRI = \lpar k_1\comma \; k_2\comma \; \ldots \comma \; k_n\rpar \eqno\lpar 10\rpar $$
  The original message can be computed from the ciphertext as
  (11) $$msg = k_1ct_1 + k_2ct_2 + \cdots + k_nct_n\bmod \lpar m\rpar \eqno\lpar 11\rpar $$

### 4.3 Proposed computationally efficient mutual authentication protocol

The proposed mutual authentication protocol constructed based on new encryption scheme. It provides mutual authentication between an infant incubator monitoring system and users. It uses ANSI/IEEE 1073: Medical Information Bus (MIB) for communication between the incubator and users. Normally, the encryption scheme is used to send the credentials to the users for checking the legitimacy. The proposed authentication protocol is an *n*-round protocol. The number of rounds depends on the security level and system parameters of the encryption method.

The new encryption scheme can be used for a different set of parameters. But, large parameter value needs more computational cost and more storage space. In medical applications, resource-limited wireless medical sensors are used to transmit the sensed information. Therefore, it is necessary to use computationally efficient protocol for transmitting the medical data without compromising the security level. Small parameters can be used instead of large parameters to reduce the computational cost and storage space.

To overcome the above problem, large size messages are separated into small pieces and every small massage is encrypted by using a new encryption scheme with the help of the receiver's public key. The proposed mutual authentication scheme consists of four essential algorithms such as a system setup algorithm, key generation algorithm, initialisation algorithm and authentication algorithm.

- *System setup algorithm:* The algorithm considers the security parameters ‘*δ*’ as an input and it gives system parameters as the output. The output parameters are $m\comma \; n\comma \; o\comma \; x\comma \; y\comma \; {\bi z}\comma \; N$ and $msg_{\max }$, where $n\comma \; N$ denotes the number of rounds and nonce used in the encryption scheme.
- *Key generation algorithm:* The algorithm considers the system output parameters as an input and it generates the pair of private and public keys as an output $\lpar PRI\comma \; PUB\rpar $. The private and public keys are
  (12) $$\lpar PRI\comma \; PUB\rpar = \lpar \lpar k_1\comma \; k_2\comma \; \ldots \comma \; k_n\rpar \comma \; \lpar PUB_1\comma \; PUB_2\comma \; \ldots \comma \; PUB_z\rpar \rpar \eqno\lpar 12\rpar $$
- *Initialisation algorithm:* To create a session between the infant incubator monitoring system and users, the algorithm considers the credential of the users (e.g. users ‘*A*’ and ‘*B*’) and the public keys $\lpar PUB_A\comma \; PUB_B\rpar $ of the users as an input. It exchanges the credentials, the public keys of the users in a secure manner by using a secure socket layer.
- *Authentication algorithm:* The mutual authentication process of users ‘*A*’ and ‘*B*’ as follows:

User ‘*A*’ randomly chooses nonce $N_{A1}$. It encrypts the message $msg_A$ by using the public key $PUB_B$ of user ‘*B*’ and it gives ciphertext as output $\lpar CT\rpar $,

(13) $$CT_1 = e_{PUB_B}\lpar msg_A\comma \; N_{A1}\rpar \eqno\lpar 13\rpar $$

After that, the user ‘*A*’ sends this ciphertext to the user ‘*B*’.

Once the ciphertext received by the user *B*, it tries to decrypt it by using its own private key $\lpar PRI_B\rpar $.

(14) $$\overline {msg_A} \comma \; N_{A1} = d_{PRI_B}\lpar CT_1\rpar \eqno\lpar 14\rpar $$

If $\overline {msg_A} = msg_A$, then it accepts the message, otherwise it rejects and terminates the connection. User *B* randomly chooses the nonce $N_{B1}$ and generate the ciphertext as

$$CT_2 = e_{PUB_A}\lpar N_{B1}\comma \; msg_B \times N_{A1}\rpar $$

Then the user *B* sends this ciphertext to the user *A*.

Once the ciphertext received by the user *A*, it decrypts the $CT_2$ by using its own private $PRI_A$ key, i.e.

(16) $$\overline {msg_B} \comma \; N_{B1} = d_{PRI_A}\lpar CT_2\rpar \eqno\lpar 16\rpar $$

**Fig. 2:**
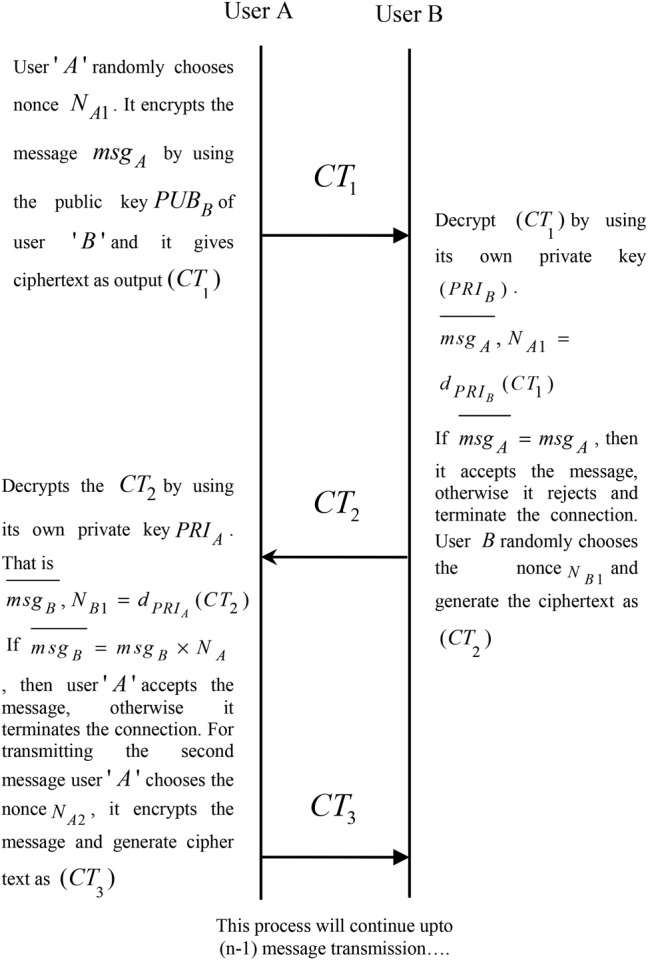
Computationally efficient mutual authentication protocol

If $\overline {msg_B} = msg_B \times N_A$, then the user ‘*A*’ accepts the message, otherwise it terminates the connection. For transmitting the second message user ‘*A*’ chooses the nonce $N_{A2}$, it encrypts the message and generates ciphertext as

(17) $$CT_3 = e_{PUB_B}\lpar msg_A \times N_{B1}\comma \; N_{A2}\rpar \eqno\lpar 17\rpar $$

and user ‘*A*’ sends this ciphertext to the user ‘*B*’. This process continues up to $n - 1$ message transmission; the functions of the proposed protocol are explained in Fig. 2.

## 5 Computational cost analysis

The proposed computationally efficient mutual authentication protocol is an *n*-round protocol. The value of *n* depends on the level of security and parameters of the new encryption scheme. If the mutual authentication protocol wants to give 32-bit security during mutual authentication, then the users need to choose the random nonce $N_A\comma \; N_B$, i.e. $\log N_A = \log N_B = 32$.

The proposed protocol runs three-round for mutual authentication. Consider the length of the user credential is *L* bits. For the three-round mutual authentication process, the length of text (original message, credentials) to be transferred is at least 32 + *L* bits. To increase computational efficiency, the message is separated into *N* pieces. For a 32-bit message, a user needs to choose four nonces $N_1\comma \; N_2\comma \; N_3$ and $N_4$, i.e. the length of the nonce is 8-bit. The users *A* and *B* need a nine-round message transmission for mutual authentication. The relationship between the number of rounds and nonce is $n = 2N + 1$.

In the three-round mutual authentication protocol, the user tries to decrypt the entire information. Also, the proposed mutual authentication protocol identifies the invalid information and it rejects, terminates the connection, and due to this, it saves energy.

## 6 Security analysis

*Key security:* The public key should be announced to the public for receiving information from the other users. But, the private key should be stored securely by the user. Once, the intruders break the encrypted information; it is easy to find the private key of the user. The opponent may try to recover the private key of a specific user from the key generation algorithm. But, due to the LWE problem, it is very hard to recover the private key.

The public key *PUB* consists of *zn*-dimensional vectors $\lpar PUB_1\comma \; PUB_2\comma \; \ldots \comma \; PUB_n\rpar $. A public key vector $PUB_i$, where $i = 2\comma \; \ldots \comma \; n\comma \; $ consists of little bit noises $\vartheta $. Due to the hardness of the LWE problem, the private key is hidden by using small noises. The private key $PRI = \lpar k_1\comma \; k_2\comma \; \ldots \comma \; k_n\rpar $. Let the rift values *R* such that $\left\vert R \right\vert = n$ of a *PUB*. It should contain minimum $2n - 1$ unknown values.

In the *n* private key values, there are $n - 1$ small noise values from the rift *R*. That is, each private key is related to the value of rift values *R*. Recovering private key value is not an easy process, because equations in a system cannot be solved individually. Therefore, the private key used in the new encryption scheme is very secure and the level of security is $\log R^{n - 1}$ bits.

Computationally efficient mutual authentication protocol security: The mutual authentication protocol is functioning based on the new encryption scheme. Therefore, the security level of the mutual authentication protocol depends on the new encryption scheme. From the key security analysis, the proposed new encryption scheme is very secure. Therefore, the proposed *n*-round mutual authentication protocol is also secure. Also, the proposed mutual authentication protocol defends against a man-in-the-middle attack and impersonation attack.

## 7 Public key size and ciphertext size optimisation

Let the public key vector

(18) $$PUB_i = \lpar PUB_{i\comma 1}\comma \; PUB_{i\comma 2}\comma \; \ldots \comma \; PUB_{i\comma n}\rpar \eqno\lpar 18\rpar $$

Public key vector elements are sampled randomly. It permits the other public key vectors to reuse the values in this public key vector. If the values of the public key $\lpar PUB_{i\comma 1}\comma \; PUB_{i\comma 2}\comma \; \ldots \comma \; PUB_{i\comma n}\rpar $ are derived from the other public key vectors, then it is enough to store the last value $PUB_{i\comma n}$ in the public key vector $PUB_i$. Consider

(19) $$\eqalign{& PUB_i = \lpar PUB_{i\comma 1}\comma \; PUB_{i\comma 2}\comma \; \ldots \comma \; PUB_{i\comma n}\rpar \quad {\rm and} \cr & PUB_j = \lpar PUB_{\,j\comma 1}\comma \; PUB_{\,j\comma 2}\comma \; \ldots \comma \; PUB_{\,j\comma n}\rpar } \eqno\lpar 19\rpar $$

are the two public key vectors. The elements of $PUB_i$ sampled randomly from $1$ to $m - 1$. To generate another public key vector $PUB_j$, the first $n - 1$ elements of $PUB_i$ vector are shifted by one position. That is a public key vector $PUB_j$ is derived as

(20) $$PUB_j = \lpar PUB_{i\comma 2}\comma \; PUB_{i\comma 3}\comma \; \ldots \comma \; PUB_{i\comma n - 1}\comma \; PUB_{i\comma 1}\comma \; PUB_{\,j\comma n}\rpar \eqno\lpar 20\rpar $$

Here, the element of $PUB_{\,j\comma n}$ can be calculated from the given $PUB_j = \lpar PUB_{\,j\comma 1}\comma \; PUB_{\,j\comma 2}\comma \; \ldots \comma \; PUB_{\,j\comma n - 1}\rpar $. Elements in the vectors can be reused maximum $n - 1$ times to avoid the situation such that two public key vectors should not have similar first $n - 1$ elements. The public key vector $PUB_i$ and $PUB_j$ are defined as $\lpar PUB_{i\comma 1}\comma \; PUB_{i\comma 2}\comma \; \ldots \comma \; PUB_{i\comma n - 1}\comma \; PUB_{i\comma 1}\comma \; PUB_{\,j\comma n}\rpar $. By using this scheme, the size of the public key can be reduced. During the implementation of this optimised public key vector, the size of the key is 68% less than the original public key vector.

In the proposed new encryption scheme, the reduced size public key is used. Therefore, the size of the output of the encryption scheme (ciphertext) is also reduced. When compared to the original ciphertext, the output of the proposed encryption scheme ciphertext size is reduced by 22 bytes. The level of security for the proposed encryption scheme is also improved because the first public key vector is secret for any equation, *n*-equation derived from the public key vectors of $2n$ unknown variables. But, there are $2n - 1$ variables in the original public key vectors.

## 8 Performance analysis of proposed computationally efficient mutual authentication protocol

To analyse the performance of the proposed mutual authentication protocol, a Cooja simulator tool (network simulator tool, specially designed for wireless sensor networks) is used, and it is implemented by using the Contiki operating system (used for low power sensor devices) [19], it is particularly used for resource limited devices. The proposed mutual authentication protocol is implemented by the C programming language. During the implantation, to increase computational efficiency, information is separated into small pieces. The settings of system parameter achieve 64-bit information security with minimum 143-bit key security and protocol security of 64 bits. As per the proposed encryption scheme, it selects an 8-bit length nonce. Therefore, it requires 17 rounds to achieve the security level of 64 bits.

During the analysis of the proposed mutual authentication protocol, the security level is 64 bits. In the original protocol, the new encryption scheme consumes 33 ms, but the optimised encryption method consumes only 27 ms. The decryption time of both the original scheme and optimised scheme are the same. The size of the ciphertext in the optimisation scheme is reduced to 176 bits when compared to the original scheme. Mutual authentication between the users is also reduced to 132 ms when comparing the optimised scheme with the original scheme. The performance comparison is shown in Table 1. During the mutual authentication between the users, the encryption scheme needs to run *n*-rounds. The number of rounds depends upon the particular message size and the size of the nonce. If the length of the nonce is 8 bits and the message size is 64 bits, then the protocol needs to run 17 rounds to complete mutual authentication, as shown in Table 2.

**Table 1:** Performance of the proposed mutual authentication protocol

Name of the protocol	Encryption time, ms	Decryption time, ms	Ciphertext size, bits	Mutual authentication time, ms
original mutual authentication protocol	33	2	446	1533
optimised mutual authentication protocol	27	2	270	1397

**Table 2:** Performance of the proposed encryption scheme

Name of the protocol	Encryption	Decryption
original mutual authentication protocol	$\lpar xn + x - 1\rpar msg + R + nM$	$\lpar n - 1\rpar msg + nM$
optimised mutual authentication protocol	$\lpar xn + x - n - 1\rpar msg + R$	$n \times msg + nM$

Table 3 shows the comparison of the Rivest–Shamir–Adleman (RSA) scheme (the oldest, secured methods of cryptographic and already in use in most of the application globally) elliptic-curve cryptography (ECC) scheme (more secured technique to enhance its security for every user) [20] and proposed scheme performance. Mutual authentication delay is reduced by 88 times when compared to the RSA scheme, and delay is reduced by 7 times when compared to the ECC scheme.

**Table 3:** Performance comparison of the proposed scheme with other existing schemes

Level of security	RSA scheme	ECC scheme	Proposed scheme
112	175,560	16,600	1960
144	327,620	27,040	2520

## 9 Conclusion

Mutual authentication is essential for wireless medical data transmission between the users in the infant incubator monitoring system. Also, the system needs to provide user authentication and data integrity by using limited resources. But, the conventional mutual authentication protocols are not suitable for resource limited devices. Therefore, in this Letter, a computationally efficient, new encryption scheme based mutual authentication protocol is presented to improve the computational efficiency with balanced information security by using limited resources in the system. The security analysis and performance analysis of the proposed computationally efficient mutual authentication protocol ensure that the proposed system outperforms when compared to other conventional mutual authentication schemes.

## References

[1] Sowmiya S. Smrithi V. Irin Loretta G.: ‘Monitoring of incubator using IoT’, Int. Res. J. Eng. Technol., 2018, 5, (4), pp. 635–638

[2] Stanford Children's Health: Available at https://www.stanfordchildrens.org/en/topic/default?id=prematurity-90-P02401, Accessed October 2018

[3] Preterm birth, Available at https://www.who.int/news-room/fact-sheets/detail/preterm - birth, Accessed September 2018

[4] Ishak D.N.F.M. Jamil M.M.A. Ambar R.: ‘Arduino based infant monitoring system’, IOP Conf. Series: Mater. Sci. Eng., 2017, 226, (1), pp. 1–6

[5] Antonucci R.: ‘The infant incubator in the neonatal intensive care unit: unresolved issues and future developments’, J. Perinat. Med., 2010, 37, (6), pp. 587–598 10.1515/JPM.2009.109 19591569

[6] Otalora A.S. Molano C.A.Q. Tovar O.M.L.: ‘Design and implementation of a prototype for neonatal intensive care incubator with fuzzy controller’, ARPN J. Eng. Appl. Sci., 2013, 8, (8), pp. 677–686

[7] Degorre C. Decima P. Degrugilliers L.: ‘A mean body temperature of 37degrees C for incubated preterm infants is associated with lower energy costs in the first 11 days of life’, Acta Paediatric., 2015, 104, (6), pp. 581–588 (doi: 10.1111/apa.12965) 10.1111/apa.12965 25661668

[8] Sawand A. Djahel S. Zhang Z.: ‘Toward energy-efficient and trustworthy e-health monitoring system’, Chin. Commun., 2015, 12, (1), pp. 46–65 (doi: 10.1109/CC.2015.7084383)

[9] Sathya D. Ganeshkumar P.: ‘Secured remote health monitoring system’, Healthc. Technol. Lett., 2017, 2, (1), pp. 1–5 10.1049/htl.2017.0033 PMC5761311 29383257

[10] Murawski K.: ‘New vision sensor to measure gas pressure’, Meas. Sci. Rev., 2017, 15, (3), pp. 132–138 (doi: 10.1515/msr-2015-0020)

[11] Salai Selvam V. Shenbaga Devi S.: ‘Analysis of spectral features of EEG signal in brain tumor condition’, Meas. Sci. Rev., 2015, 15, (4), pp. 219–225 (doi: 10.1515/msr-2015-0030)

[12] Shin M.S. Jeon H.S. Ju Y.W.: ‘Constructing RBAC based security model in u-healthcare service platform’, Scientific World J., 2015, 4, (3), pp. 456–461 10.1155/2015/937914 PMC4322305 25695104

[13] Wang A. Zhang B. Ren K.: ‘A privacy-aware cloud-assisted healthcare monitoring system via compressive sensing’. Proc. of 33rd IEEE INFOCOM, Toronto, ON, Canada, 2014, pp. 2130–2138

[14] Nachabe L. Marc C. Bachar A.: ‘M-health application for neonatal incubator signals monitoring through a CoAP-based multi-agent system’. Int. Conf. on Advanced Research in Business, Management and Economics (ICABME15), Beirut, Lebanon, 2015, pp. 1–5

[15] Bekara D. Laurent-Maknavicius M.: ‘A new protocol for securing wireless sensor networks against nodes replication attacks’. Proc. of 3rd IEEE Int. Conf. on Wireless and Mobile Computing, Networking and Communications (WiMOB 2007), White Plains, NY, USA, 2007, pp. 59–59

[16] Sivasankar P.T. Ramakrishnan M.: ‘Active key management scheme to avoid clone attack in wireless sensor network’. Proc. of 4th Int. Conf. on Computing, Communications and Networking Technologies (ICCCNT'13), Tiruchengode, India, 2013, pp. 1–4

[17] Marcos A. Simplicio J. Leonardo H.I.: ‘Secourhealth: a delay-tolerant security framework for mobile health data collection’, IEEE J. Biomed. Health Inf. (IEEE Trans. Inf. Technol. B), 2015, 19, (2), pp. 761–772 10.1109/JBHI.2014.2320444 24801629

[18] He D. Zeadally S. Kumar N.: ‘Anonymous authentication for wireless body area networks with provable security’, IEEE Syst. J., 2017, 11, (4), pp. 2590–2601 (doi: 10.1109/JSYST.2016.2544805)

[19] ‘Programming Internet of Things using Contiki and Cooja’, Available at https://opensourceforu.com/2017/06/programming-internet-things-using-contiki-cooja, Accessed November 2018

[20] Bhujade V. Chaudhary D.: ‘Analysis and implementation of combined approach of RSA and ECC algorithm for enhanced data security’, Int. J. Comput. Appl., 2015, 129, (15), pp. 37–42

